# Supplementary material for: Molecular Effect of Variants in Toll-like Receptor 4 Gene in Saudi Patients with Type 2 Diabetes Mellitus
Source: Cells. 2023 Sep 23;12(19):2340. doi: 10.3390/cells12192340 (PMC10571932; doi:10.3390/cells12192340)

Figure S1. Presentation of CC, CT and TT genotypes appears in rs4986791 SNP using in 3% agarose gel.

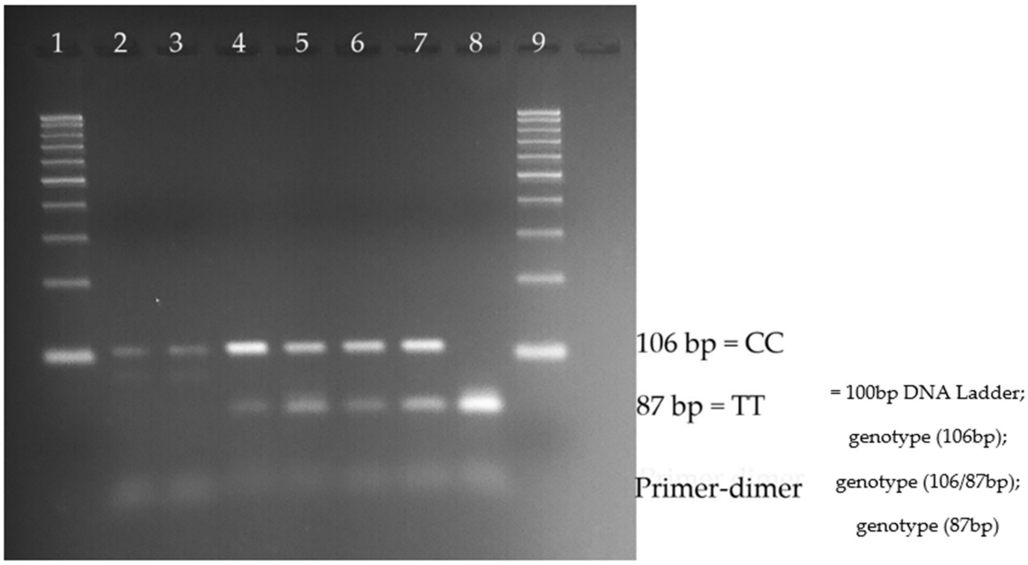

106 bp = CC

87 bp = TT

Primer-dimer

Lane 1 and 9 = 100bp DNA Ladder;

Lane 2-3: CC genotype (106bp);

Lane 4-7: CT genotype (106/87bp);

Lane 8: TT genotype (87bp)

Table S1. List of *TLR4* SNPs and primer details are presented in this study.

Position	rs number	SNP	Location	Forward Primer	Reverse Primer	PCR Size	Annealing Temperature	Restriction Enzyme	Digested Products
G7263C	rs11536889	G-A	3′UTR	GCAGGAAGGAAGTGGGATGAC	TGTTTCTGAGGAGGCTGGATG	391bp	64^{0}C	-	-
A896G	rs4986790	A-G	Exon-3	AGCATACTTAGACTACTACCTCCATG	GAGAGATTTGAGTTTCAATGTGGG	200bp	56^{0}C	-	-
C1196T	rs4986791	C-T	Exon-3	CTCAAAGTGATTTTGGGAGAA	AGATGTTCTAGTTGTTCTAAGCC	106bp	60^{0}C	HinfI (G↑AATC)	C-106bp; T-87/19bp

Figure S2. Performed validation for rs11536889 and rs4986790 SNPs via Sanger sequencing.

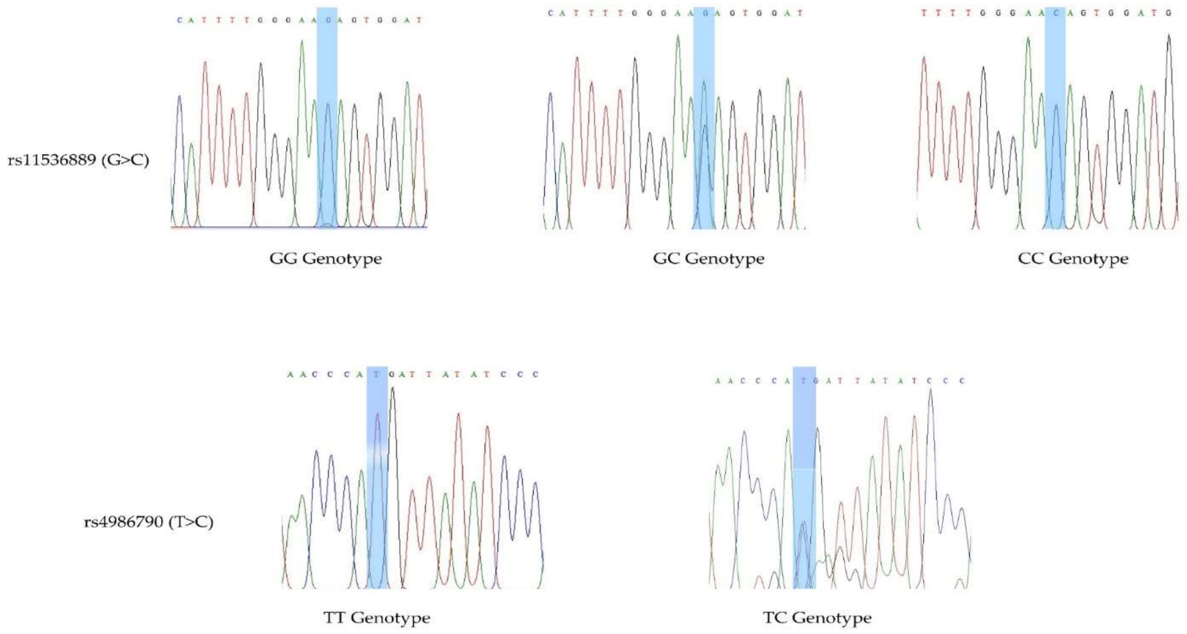

Supplement: Supplementary file 1 [file cells-12-02340-s001.zip › cells-2581187-supplementary.pdf]
